# Poly(Glycerol‐Sulfur) as a Functional Sustainable Nanomaterial: Synthesized by Anionic Ring Opening Polymerization of Elemental Sulfur

**DOI:** 10.1002/smll.74499

**Published:** 2026-07-11

**Authors:** Paraskevi S. Stergiou, Mariam Cherri, Philip Nickl, Elisa Quaas, Katharina Achazi, Mathias Dimde, Mohsen Adeli, Rainer Haag

**Affiliations:** ^1^ Institute of Chemistry and Biochemistry Freie Universität Berlin Takustr Berlin Germany; ^2^ Institute of Chemistry and Biochemistry Research Center of Electron Microscopy Freie Universität Berlin Berlin Germany; ^3^ Department of Organic Chemistry, Faculty of Chemistry Lorestan University Khorramabad Iran

**Keywords:** poly(glycerol‐sulfur), redox‐responsiveness, sulfur copolymerization, sustainable sulfur copolymers, thiol–disulfide exchange reaction

## Abstract

Elemental sulfur is an abundant by‐product of the petroleum industry, yet its controlled incorporation into functional polymer architectures remains challenging. Here, we report a one‐pot, moderate‐temperature (120°C) anionic copolymerization of elemental sulfur (S_8_) with glycidol to produce hyperbranched poly(glycerol‐sulfur)s with tunable molecular weights (5–30 kDa) and sulfur contents (3–11 wt%) on the gram scale. The reaction proceeds under markedly milder conditions than conventional sulfur polymerizations, enabling precise control over polymer structure and programmable, redox‐triggered backbone cleavage rates in glutathione‐rich environments. Comprehensive spectroscopic characterization and control experiments provided mechanistic insights into the copolymerization of S_8_ and glycidol. The method is readily scalable to tens of grams and affords structural precision unattainable by high‐temperature radical routes. As a proof of concept, the copolymers were functionalized with the cytotoxic DM1 via thiol–disulfide exchange, yielding nanocarriers that release over 90% of the payload within 12 h under reductive conditions and significantly reduced the viability of MCF7 breast cancer cells.

## Graphical abstract

Petroleum‐derived waste sulfur is transformed into functional hyperbranched copolymers through a scalable one‐pot polymerization with glycidol. The resulting water‐soluble, redox‐responsive nanomaterials exhibit tunable structures and enable controlled release applications for advanced therapeutic delivery.

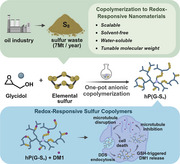

## 1 Introduction

Elemental sulfur is one of the most abundant elements on Earth and is naturally present in large quantities due to volcanic activity, natural gas processing, and petroleum refining [1]. These industrial processes have led to a significant surplus of sulfur worldwide, creating both a storage challenge and an opportunity for sustainable innovation [2, 3]. Therefore, utilizing sulfur in functional materials such as polymers has become a focus of research [4, 5, 6]. Although the synthesis of sulfur‐containing polymers has primarily relied on radical polymerization of elemental sulfur, the high temperatures required for these reactions have motivated growing interest in anionic polymerization as a milder and more controlled synthetic alternative [3, 7, 8, 9, 10, 11, 12]. Sulfur's unique redox chemistry, high polarizability, and capacity to form dynamic covalent S–S*_n_* linkages make it an attractive building block for applications in energy storage, sensing, environmental remediation, and responsive biomedical systems [13, 14, 15, 16, 17, 18]. However, the preparation of sulfur‐containing polymers with controlled composition and architecture remains limited by the poor compatibility of S_8_ with many polymerization methods, owing to its high ring stability which requires significant energy input for opening, and poor solubility in most organic solvents [19, 20, 21].

Synthetic advances, including inverse vulcanization and thiol‐ene chemistry, have expanded access to sulfur‐rich polymers; yet most approaches rely on high‐temperature, free‐radical processes that afford limited control over molecular weight, dispersity, and branching [22, 23, 24, 25]. Moreover, these methods often restrict monomer scope and lack tunability in the sulfur content and chain microstructure‐parameters that critically influence redox behavior and functional performance across application areas [26, 27]. A low‐temperature, selective, and scalable method for incorporating elemental sulfur into defined polymer architectures would therefore open new opportunities for designing multifunctional, stimuli‐responsive material platforms.

In this work, we report a one‐pot anionic copolymerization of elemental sulfur (S_8_) and glycidol conducted at moderate temperatures (120°C), significantly reducing the thermal input compared to conventional inverse vulcanization (>159°C). This approach yields hyperbranched poly(glycerol‐sulfur) with tunable molecular weight, branching, and sulfur content. A mechanistic investigation led to the proposal of a reaction pathway toward the product, whose inherent polysulfide bonds enable facile post‐modification via thiol–disulfide exchange. By tuning molecular weight, branching, and sulfur content, the composition of these copolymers can be directly correlated with their redox‐triggered degradation behavior, thereby establishing a versatile platform for designing sulfur‐containing copolymers with broad functional potential. In particular, biodegradable and redox‐responsive carriers are attractive for drug delivery, as the high intracellular glutathione concentration in tumor cells can selectively trigger release of thiol‐linked therapeutics [28, 29, 30]. As a proof‐of‐concept, we conjugated the potent anticancer agent DM1 to the hyperbranched poly(glycerol‐sulfur) copolymers via thiol–disulfide exchange, enabling redox‐triggered release in vitro. This work highlights the potential of these copolymers as responsive nanocarriers, while underscoring their broader promise as sustainable and versatile functional polymer platforms.

## 2 Experimental

Details regarding materials, methods, synthesis, and characterization can be found in ESI.

## 3 Results and Discussion

**FIGURE 1:**
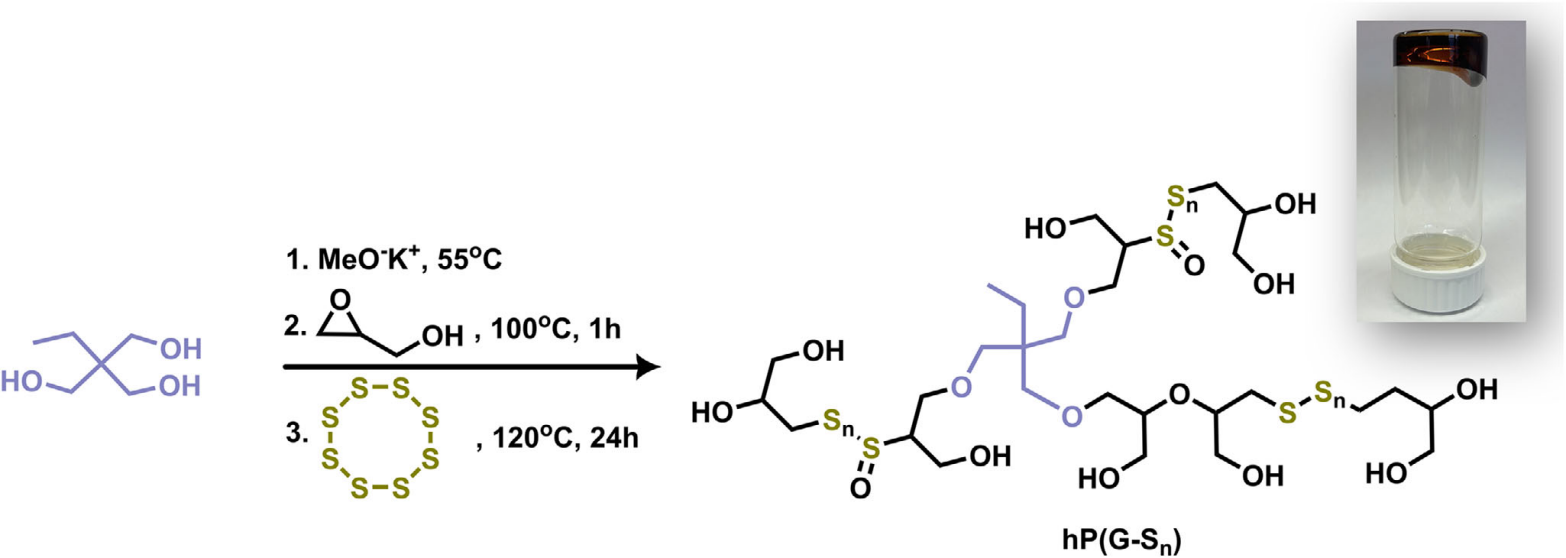
Schematic representation of the anionic copolymerization of glycidol and elemental sulfur (S_8_) to produce hP(G‐S*_n_*).

Elemental sulfur was copolymerized with glycidol via anionic ring‐opening polymerization, using trimethylolpropane (TMP) as an initiator (Figure 1). The anionic polymerization of glycidol initiated by TMP is a well‐established procedure and results in hyperbranched polyglycerols with different molecular weights, based on the quantity of initiator [31]. For the copolymerization of glycidol and S_8_, glycidol was added to the deprotonated TMP and then mixed with the melted elemental sulfur. More specifically, TMP was deprotonated following the procedure reported by Sunder et al. [32] after which glycidol was added dropwise over 2 h at a rate of 2.5 mL min^−^^1^ at 70°C. After 1 h of glycidol addition, S_8_ was added to the mixture and stirred overnight at 120°C. hP(G‐S*_n_*) was obtained as a homogeneous amorphous brown viscous compound, after purification (Figure 1). Since pure polyglycerol is a colorless viscous compound, the brown color was the first optical indication of sulfur's incorporation in the polymer structure.

The copolymers were denoted as hP(G*_x_*‐S*_y_*)*_z_*, where *x* represents the targeted mol% of glycidol, *y* the targeted mol% of S_8_, and *z* the targeted molecular weight of the copolymer in kilodaltons (kDa). Overall, five types of copolymers were synthesized by either targeting the same molecular weight and varying the sulfur content or keeping the same sulfur content and varying the molecular weight. To demonstrate reaction scalability, we performed a 50‐gram scale‐up reaction that yielded a product with the same properties as the gram‐scale material. The composition and molecular characteristics of the synthesized copolymers are shown in Table 1.

**TABLE 1:** Copolymer compositions and properties, including monomer feed ratios, targeted and calculated molecular weights, sulfur content, and scale‐up reaction data.

	[Gly]:[S_8_]	MW^a^ (kDa)	MW^b^ (kDa)	S content^c^ (%)	Yield (%)
hP(G_95_‐S_5_)_5_	95:5	6	6.3	1.3	79
hP(G_85_‐S_15_)_5_	85:15	6	5.2	3.9	78
hP(G_50_‐S_50_)_5_	50:50	6	4.4	10.9	30
hP(G_50_‐S_50_)_10_	50:50	12	9.4	11.1	39
hP(G_50_‐S_50_)_20_	50:50	25	22.6	15.9	25
hP(G_85_‐S_15_)_40_ ^d^	85:15	40	39.7	3.4	47

^a^ Targeted molecular weight.

^b^ Molecular weight measured by ^1^H NMR.

^c^ Sulfur content measured by elemental analysis.

^d^ Scale‐up reaction.

From Table 1, it is evident that the isolated yields decrease with increasing sulfur feed ratio, while the experimentally determined sulfur content remains significantly below the theoretical values based on the initial monomer composition. This behavior indicates that sulfur incorporation does not proceed in a statistical manner with respect to the feed composition. Additionally, the formation of sulfur‐rich by‐products and the removal of unreacted sulfur during purification contribute to the reduced isolated yields.

**FIGURE 2:**
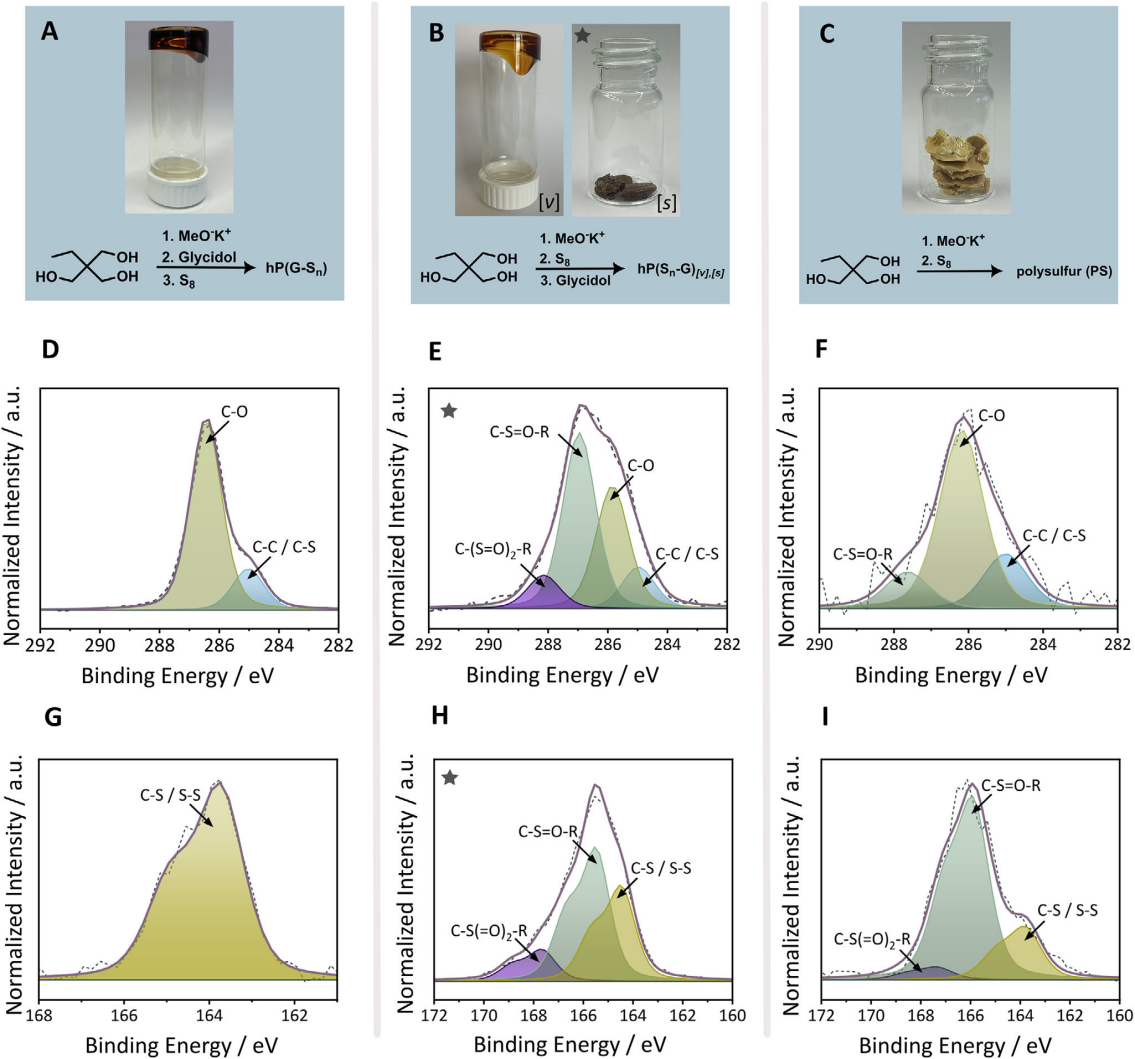
(A) Reaction conditions of the synthesis of hP(G_85_‐S_15_)_5._ A viscous dark brown compound was obtained. (B) Reaction conditions of the synthesis of hP(S*_n_*‐G). Two different products, a brown viscous and a dark solid compound, were obtained. (C) Reaction between deprotonated TMP and elemental sulfur resulted in a yellow solid compound. (D,E,F) C 1s XP spectra of hP(G_85_‐S_15_)_5_, hP(S*_n_*‐G)_solid_, and PS demonstrating C–C, C–S, and C–O peak components. (G,H,I) S 2p XP spectra of hP(G_85_‐S_15_)_5_, hP(S*_n_*‐G)_solid_, and PS indicating C–S and S–S as the main bonds in the structure of these materials and small oxidized sulfur contents for the two latter.

To investigate the proper order of monomer addition, a control reaction was performed, in which sulfur was first added to deprotonated TMP and melted, followed by the controlled addition of glycidol at a rate of 2.5 mL min^−1^. The reaction mixture was then stirred overnight at 120°C. This reaction yielded two products: a brown solid (hP(S‐G)_solid_) and a brown‐red viscous (hP(S‐G)_viscous_) compound (Figure 2B). The observed heterogeneity of the reaction products suggested that this method was less suitable for uniform copolymer formation than the first approach.

To determine the reactivity of sulfur and the mechanism of copolymerization, a second control experiment was performed. In this reaction, S_8_ was added to deprotonated TMP and stirred overnight at 120°C. A yellow solid product, denoted as polysulfur (PS), was obtained. This reaction confirmed the reactivity of elemental sulfur against alkoxides under the specified conditions (Figure 2C). This result suggested that deprotonated TMP can initiate the ring‐opening polymerization of elemental sulfur, producing thiolates that can be further copolymerized with glycidol via an anionic ring‐opening mechanism.

The structures of hP(G_85_‐S_15_)_5_, as well as the products of the two control reactions, hP(S‐G)_solid_ and PS, were analyzed by X‐ray photoelectron spectroscopy (XPS) (Figure 2). Survey XP spectra revealed carbon, sulfur, and oxygen as the main components of all the aforementioned compounds, as expected (Figure S2, Table S2). The elemental composition (%) of the different products showed sulfur contents of 3.6% for hP(G_85_‐S_15_)_5_, 30.7% for hP(S‐G)_solid_, and 63.4% for PS, all aligning with the trend expected from the reaction conditions, with hP(G_85_‐S_15_)_5_ showing lower sulfur incorporation and the control reactions yielding sulfur‐richer products. The highly resolved C 1s spectra of hP(G_85_‐S_15_)_5_ (Figure 2D) displayed a small peak component at 285 eV, which was attributed to C–C bonds of TMP and C–S bonds of the copolymer's backbone, and the main peak at 286.4 eV corresponding to C–O bonds of the copolymer's backbone. This spectroscopy data confirmed the initiation of glycidol polymerization by TMP, followed by the incorporation of S_8_ into the copolymer backbone through reaction with alkoxides. In the highly resolved C 1*s* spectra of hP(S‐G)_solid_ (Figure 2E), peaks at 285 eV, 286 eV, and 287 eV were assigned to C–C/C–S, C–O, and C–S(=O)–R bonds, respectively [33, 34]. The presence of C–C and C–S bonds in hP(S‐G)_solid_ indicated opening of the S_8_ ring by TMP and then partial polymerization of glycidol monomers by generated thiolate groups. However, in the case of PS (Figure 2F), glycidol was not added to the reaction; yet a peak at 286 eV corresponding to C–S bonds was observed. This suggested that the alkoxide of TMP nucleophilically attacks the ring of S_8_, forming C–S–O bonds that undergo intramolecular rearrangement to form more stable C–S(=O) bonds. In the highly resolved S 2p spectra of hP(G_85_‐S_15_)_5_ (Figure 2G) one peak at 164 eV corresponding to C–S and S–S bonds was observed. This further indicated the incorporation of S_8_ in the structure of hPG. In the S 2p spectra of hP(S‐G)_solid_ and PS (Figures 2H,I) distinct signals at 164 eV, 166 eV, and 167.5 eV were observed, corresponding to C–S/S–S, C–S(=O), and C–S(=O)_2_ bonds respectively. These peaks were attributed to C–S(=O) bonds formed via intramolecular rearrangement of C–S–O bonds. All XPS fitting parameters are provided in Table S3.

**FIGURE 3:**
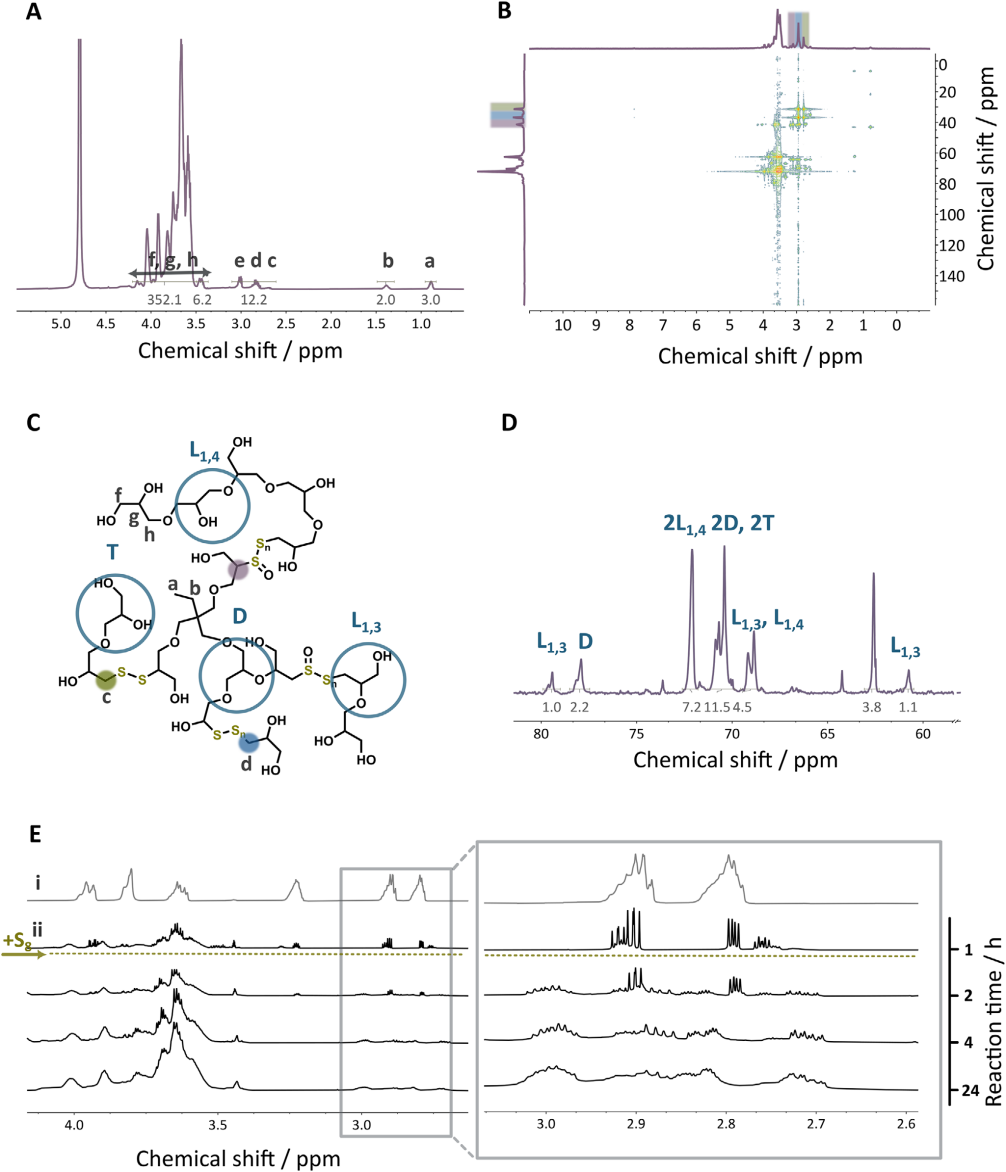
Characterization of hP(G_85_‐S_15_)_5_ by different NMR spectroscopy methods. (A) ^1^H NMR of hP(G_85_‐S_15_)_5_ indicating copolymerization of S_8_ and glycidol, due to the emerged signals between 2.7 and 3.0 ppm. (B) HMBC NMR of hP(G_85_‐S_15_)_5_ showing correlation between carbon and proton signals of methylene groups connected to polysulfide segments. (C) Chemical structure of the copolymer depicting structural units and protons correlated to ^13^C, ^1^H, and HMBC NMR signals. (D) Inverse‐gated ^13^C NMR of hP(G_85_‐S_15_)_5_ revealing the structural units and branching points of the copolymer. (E) ^1^H NMR of (i) mixture of glycidol and S_8_ at room temperature, (ii) hP(G_85_‐S_15_)_5_ at reaction times of 1, 2, 4, and 24 h, showing the disappearance of glycidol's peaks with a simultaneous appearance of peaks corresponding to integration of S_8_ in the polyglycerol's backbone.

To gain a deeper understanding of the copolymers’ structures and the reaction mechanism, hP(G*_x_*‐S*_y_*)*_z_* copolymers were characterized using various spectroscopic and microscopic techniques, along with thermal gravimetric and elemental analysis. In the ^1^H NMR spectra of hP(G_85_‐S_15_)_5_, signals at 0.9 and 1.4 ppm were assigned to the methyl and methylene groups of TMP, respectively (Figure 3A). Additional signals at 3.4–4.2 ppm were attributed to the protons of polyglycerol. Signals at 2.7, 2.8, and 3.0 ppm were assigned to the protons of the methylene groups attached to polysulfide segments (CH_2_–S*_n_*‐), indicating covalent bonds between sulfur‐containing bridges and the polyglycerol backbone. More specifically, the signal at 3.0 ppm was assigned to the methylene protons connected to oxidized sulfur, CH_2_–S(=O), which is produced by the rearrangement of CH_2_–S–O bonds, while signals at 2.7 and 2.8 ppm were attributed to methylene protons connected to disulfide and polysulfide bonds, respectively. The molecular weight of the copolymers, which was calculated using the peak area of TMP to polyglycerol backbone from the respective ^1^H NMR spectra, reveals the molecular weights of the synthesized copolymers. In all ^1^H NMR spectra, a direct correlation was observed between the peak areas of the protons of the methylene groups bonded to sulfur and the sulfur feed ratio. These results, consistent with the elemental analysis, confirmed that a higher sulfur feed results in greater sulfur incorporation in the final copolymers. In the ^13^C NMR spectra, signals at 31 and 37 ppm were attributed to the CH_2_–S*_n_*‐ groups, which are in alignment with the ^1^H NMR spectra and further support the incorporation of sulfur in the polyglycerol backbone. The signal at 41 ppm was assigned to the CH_2_–S(=O) group, formed by rearrangement of CH_2_–S–O bonds. Signals corresponding to the backbone of polyglycerol can be seen at 60–70 ppm (Figure 3B). ^1^H and ^13^C NMR spectra of the other synthesized copolymers are provided in the ESI (Figure S3 and S4).

Heteronuclear multiple bond correlation nuclear magnetic resonance (HMBC NMR) spectra of hP(G_85_‐S_15_)_5_ showed correlation between signals at 2.7, 2.8, and 3 ppm with carbon atoms at 31, 37, and 41 ppm, respectively. This correlation confirmed the assignment of signals in ^1^H NMR and ^13^C NMR spectra of hP(G_85_‐S_15_)_5_ and successful copolymerization of sulfur and glycidol (Figure 3B).

Figure 3C depicts the chemical structure of the synthesized copolymers and highlights the assigned protons to the respective signals in NMR spectra. Due to the proton transfer between the alkoxy and hydroxyl functional groups, copolymerization of glycidol and S_8_ is expected to result in hyperbranched structures. To verify this, inverse‐gated ^13^C NMR spectra of copolymers were recorded and the structural units’ relative abundance as well as the degree of branching (DB) were calculated (Figure 3D and Table S4). The copolymers demonstrated a DB in the range of 30%–50% supporting the formation of hyperbranched structures. Targeting the same molecular weight but different sulfur feed ratios in hP(G_95_‐S_5_)_5_, hP(G_85_‐S_15_)_5_, and hP(G_50_‐S_50_)_5_, almost the same DB was obtained. However, an inverse correlation between the DB and molecular weight was observed when the same sulfur feed ratios were used. These results demonstrated that DB is a function of the quantity of initiator and that the sulfur feed ratio has negligible effect on this structural parameter.

Next, a kinetic study was conducted, monitored by ^1^H NMR spectroscopy, to observe the incorporation of S_8_ into the polyglycerol backbone. Figure 3E(i) displays the ^1^H NMR spectra of a mixture of glycidol and S_8_ at room temperature, used as a reference representing the data point right after mixing glycidol and S_8_. Figure 3E(ii) reveals the signals of glycidol's protons at 2.75, 2.80, and 2.90 ppm after 1 h of reaction between TMP and glycidol, right before the addition of S_8_. At this stage, residual glycidol signals are clearly present, indicating incomplete monomer conversion. Time‐resolved ^1^H NMR integrations (Figure S3) further support this observation and indicate a progressive increase in the degree of polymerization, as determined relatively to the TMP reference signals. Although the overlap between glycidol and sulfur‐containing polymer signals in the 2.70–3.00 ppm region prevents direct quantification of glycidol consumption, the increase in integration confirms continued polymer growth beyond this point.

Upon the addition of S_8_, glycidol feeding was maintained at a rate of 2.5 mL/h for an additional hour. The appearance of new signals in the 2.70–3.00 ppm region, corresponding to methylene groups in polyglycerol units connected to polysulfide linkages, indicates incorporation of sulfur into the growing polymer. After 4 h, the reaction continues with further polymer formation, as evidenced by the increase in signals in the 2.70–3.00 ppm and 3.50–4.00 ppm regions, corresponding to polyglycerol backbone formation and sulfur‐containing units, respectively.

After 24 h, complete consumption of glycidol is confirmed by the disappearance of signals at 2.75, 2.80, and 2.90 ppm, alongside the corresponding increase in polymer signals, indicating full monomer conversion and successful incorporation of sulfur into the copolymer.

**FIGURE 4:**
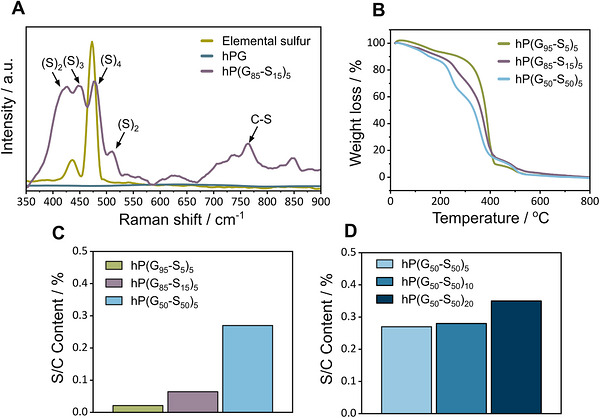
(A) Raman spectra of hP(G_85_‐S_15_)_5_, hPG, and S_8_, in the characteristic region of polysulfide bonds and C–S bonds, showing the diversity of the polysulfide segments and the presence of the C–S bonds in the copolymer's structure. (B) TGA thermograms of the copolymers synthesized by different glycidol/S_8_ feed ratios, depicting the main weight loss in the range of 270°C–400°C. (C) Elemental analysis sulfur to carbon ratios for the copolymers synthesized using different glycidol/S_8_ ratios while targeting the same molecular weight. (D) Sulfur to carbon ratios of copolymers synthesized using the same glycidol/S_8_ ratio while targeting different molecular weights.

To investigate the polysulfide segments incorporated into the structure of polyglycerol, Raman spectra of the copolymers were recorded. Figure 4A depicts the characteristic range of the Raman shift at 675–800 cm^−1^, which corresponds to the C–S bonds present in the copolymer structure [35]. The bands between 400 and 530 cm^−1^ were assigned to the stretching vibrations of the polysulfide bonds. More specifically, peaks at 435 and 516 cm^−1^ were assigned to disulfide bonds, while peaks at 458 and 485 cm^−1^ were assigned to trisulfide and tetrasulfide bonds, respectively [35, 36]. The diversity of polysulfide segments in terms of the number of sulfur atoms was attributed to the susceptibility of S–S bonds to thiol–disulfide exchange reactions that can occur via thiolate groups during the copolymerization. The intramolecular rearrangement of CH_2_–S–O to more stable CH_2_–S(=O) bonds is indicated in the ESI (Figure S5) [37].

The thermal stability of hP(G_95_‐S_5_)_5_, hP(G_85_‐S_15_)_5_, and hP(G_50_‐S_50_)_5_ was evaluated by thermogravimetric analysis (TGA). hP(G_95_‐S_5_)_5_ which has the lowest sulfur content, exhibited thermal behavior similar to hyperbranched polyglycerol with a major weight loss (∼80%) occurring at 270°C. In contrast, hP(G_50_‐S_50_)_5_, containing the highest sulfur content, demonstrated two distinct decomposition events: the first at 200°C, attributed to the degradation of the polysulfide segments, and the second at 300°C, corresponding to the decomposition of the polyglycerol backbone. The thermal degradation profile of hP(G_85_‐S_15_)_5_ fell between those of the other two copolymers, consistent with its intermediate sulfur content. These observations indicated an inverse correlation between sulfur content and thermal stability of the copolymers. The reduced thermal stability with increasing sulfur content is attributed to the higher abundance of thermally labile S–S bonds.

**FIGURE 5:**
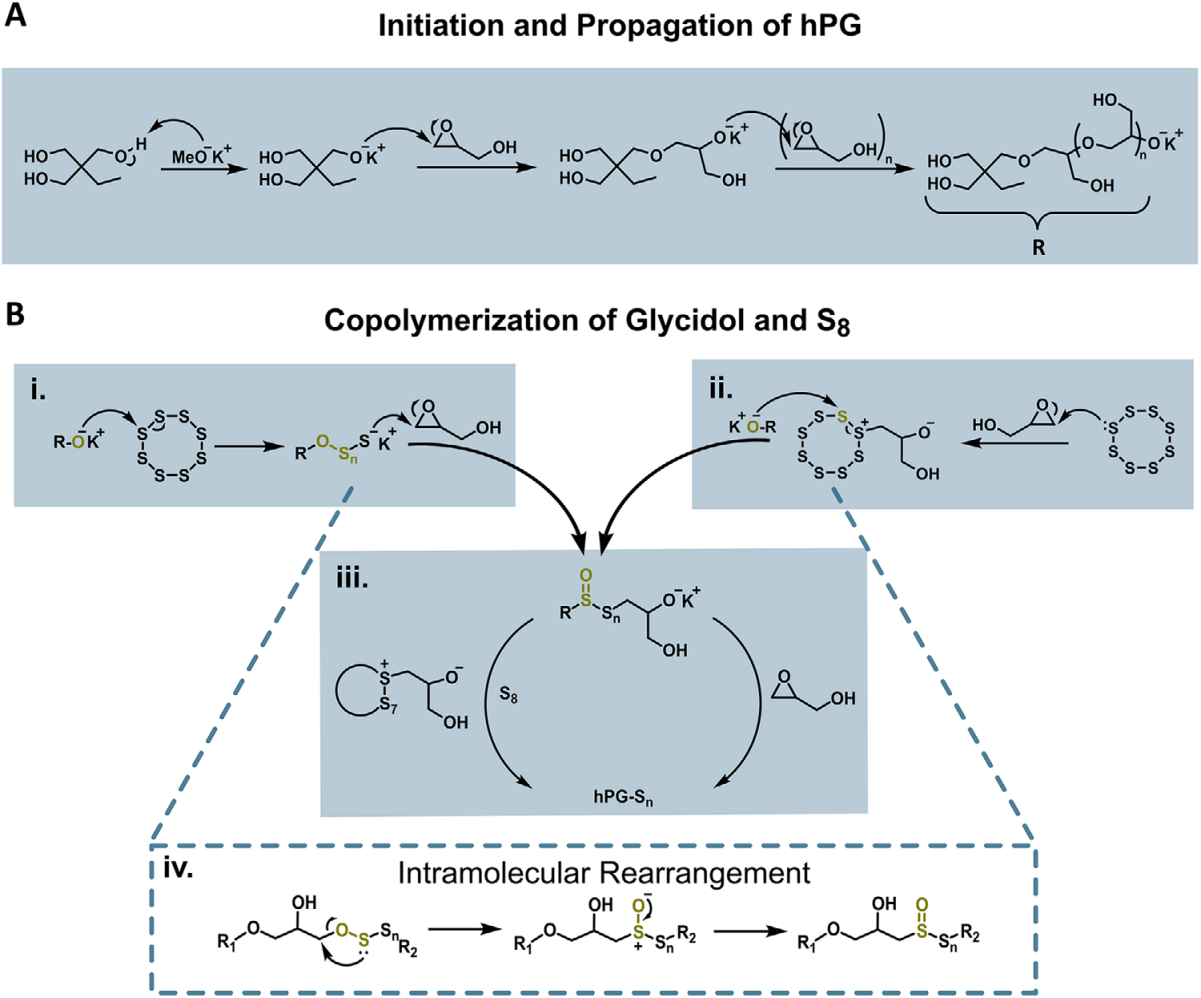
Proposed mechanism for the anionic ring opening copolymerization of glycidol and S_8_. (A) Initiation and partial propagation of hPG. (B) Nucleophilic attack of alkoxide groups to open the ring of S_8_, leading to intermediate active species (i), nucleophilic attack of S_8_ to glycidol, leading to positively charged species which is further neutralized by another alkoxide group (ii), propagation of the copolymerization (iii), and intramolecular rearrangement of the intermediate species to form more stable C–S(=O) bonds (iv).

We first investigated the incorporation of sulfur by varying the amount of sulfur while targeting the same MW. Elemental analysis revealed a monotonic increase in sulfur incorporation with increasing sulfur feed ratio is. Specifically, increasing the S_8_ content while targeting the same MW led to higher sulfur incorporation in the copolymer (Figure 4C). Mechanistically, this can be attributed to the nucleophilic attack of alkoxide groups at the active chain ends on S_8_, which subsequently allows further attack on glycidol monomers (Figure 5Bi).

Next, we examined polymerizations at varying MWs while keeping the sulfur to glycidol ratio constant. In this case, higher MWs led to increased sulfur incorporation, suggesting the dependence on the concentration of reactive chain ends (Figure 4D). This observation implies an alternative reaction pathway, in which S_8_ reacts with glycidol monomers to form reactive intermediates (Figure 5Bii). Both pathways converge at a common intermediate, which can propagate polymerization through attack on either glycidol or S_8_ monomers (Figure 5Biii). These trends are presented qualitatively, and no formal kinetic model is implied.

Support for these mechanisms was provided by ^1^H and ^13^C NMR spectroscopy. Signals at 2.7 ppm (^1^H) and 31 ppm (^13^C), corresponding to methylene groups adjacent to polysulfide units, are consistent with nucleophilic attack of S_8_ and thiolate group formation. Additional signals at 2.8–3 ppm (^1^H) and 37–41 ppm (^13^C) for methylene groups adjacent to sulfoxide groups indicated nucleophilic attack of alkoxide groups on S_8_.

On the basis of these findings, we propose a mechanism for the anionic copolymerization of glycidol and S_8_ (Figure 5). TMP is first deprotonated by potassium methoxide (MeO^−^K^+^), generating an alkoxide anion that initiates epoxide ring‐opening polymerization of glycidol (Figure 5A). After partial polymerization, S_8_ is introduced and reacts with the alkoxide‐terminated chains via ring‐opening. Once incorporated, the copolymerization propagates through successive attacks by thiolate or alkoxide groups on glycidol or S_8_ monomers and reactive intermediates. The resulting C–S–O bonds can undergo intramolecular rearrangements to form more stable C–S(=O) bonds. Consistent with the observed discrepancy between the sulfur feed ratio and incorporation, sulfur insertion proceeds via nucleophile‐activated reactions involving both alkoxide chain ends and sulfur‐derived species. Alkoxide‐mediated activation of S_8_ enables insertion at growing chain ends, while sulfur species can also react with glycidol and propagating chains. Overall, these processes result in kinetically controlled and nonstatistical incorporation relative to glycidol polymerization.

Hydrogen abstraction reactions cannot be excluded under the applied conditions, particularly in the presence of reactive sulfur species such as thiolates or polysulfide intermediates. However, based on spectroscopic analysis and the absence of significant structural irregularities or crosslinking, such processes are not expected to play a dominant role in determining the polymer structure.

Raman spectroscopy of hP(G_85_‐S_15_)_5_ showed vibrational modes in the 950–1300 cm^−1^ region, confirming the presence of sulfoxide (S = O) groups (Figure S5). Raman analysis also indicated the presence of short polysulfide segments (S_2_, S_3_, S_4_) within the copolymer structure, supporting the proposed mechanism. These short chains likely arise from chain transfer reactions between growing thiolate ends and longer polysulfide chains, as the weaker S–S bonds in longer chains are prone to cleavage [21, 38].

Among the synthesized copolymers, hP(G_50_‐S_50_)_20_ was selected for the controlled delivery of DM1, due to the high sulfur contents and higher molecular weight. The anticancer drug DM1, which contains a free thiol group, was covalently conjugated to the copolymer through a thiol–disulfide exchange reaction and the product (hP(G_50_‐S_50_)_20_‐DM1) was obtained by nanoprecipitation.

**FIGURE 6:**
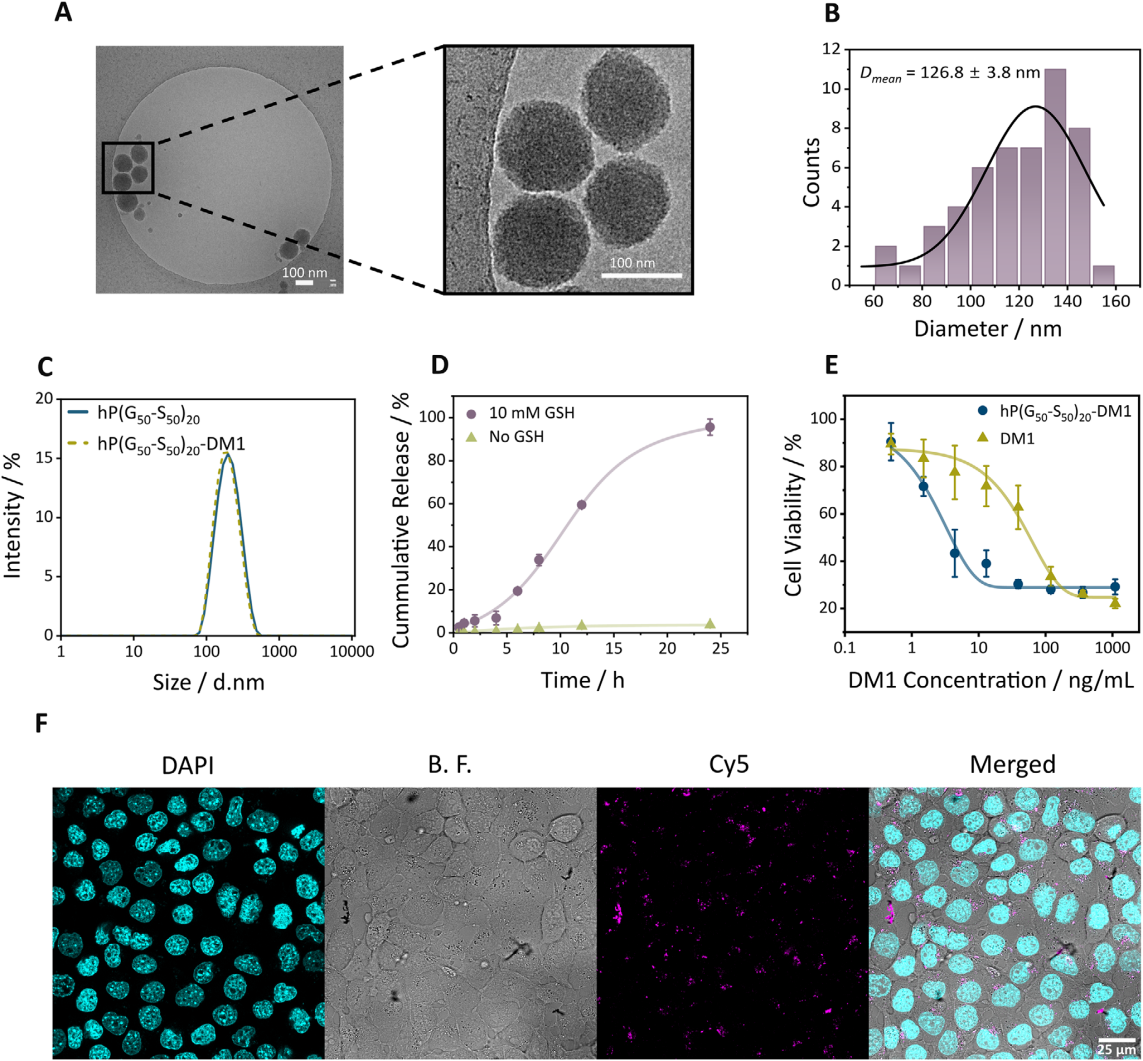
(A) Cryo‐TEM image of hP(G_50_‐S_50_)_20_‐DM1(scale bar, 100 nm). (B) Histograms of 50‐particle size from the analysis of cryo‐TEM images of hP(G_50_‐S_50_)_20_‐DM1. (C) Size distribution of hP(G_50_‐S_50_)_20_ and hP(G_50_‐S_50_)_20_‐DM1 measured by DLS. (D) In vitro release profile of hP(G_50_‐S_50_)_20_‐DM1 in the presence and absence of GSH (10mм). (E) Viability of MCF7 cells following 24 h incubation with hP(G_50_‐S_50_)_20_ ‐DM1 and DM1. (F) CLSM images of MCF7 cells incubated with hP(G_50_‐S_50_)_20_‐Cy5 (1 mg/mL) for 2 h (scale bars 25 µm). DAPI (cyan): cell nuclei; BF (grey scale): bright field image; Cy5 (magenta): copolymer [hP(G_50_‐S_50_)_20_].

Cryo‐TEM images revealed that hP(G_50_‐S_50_)_20_‐DM1 adopted a spherical morphology (Figure 6A), and analysis of 50 individual particles indicated an average diameter of 126.8 nm (Figure 6B). The hydrodynamic size and polydispersity of hP(G_50_‐S_50_)_20_‐DM1 were characterized by dynamic light scattering (DLS) which exhibited a size of 178.6 nm, similar to the size of hP(G_50_S_50_)_20_, and a PDI of 0.11 (Figure 6C). The increased size in DLS measurements was attributed to the fact that DLS provides information on the hydrodynamic diameter of the particles. HPLC measurements revealed a loading capacity (LC) of 3.3 wt% (theoretical 10%). Given DM1's high potency, this drug loading was sufficient for effective delivery. One of the main challenges in traditional drug delivery systems is the leaking and unspecific release of drugs from the carrier. By employing stimuli‐responsive drug delivery systems, such problems are mitigated. Redox‐responsive drug delivery systems with the ability of controlled cargo release, triggered by biological redox agents, are promising vectors for cancer therapy [29]. hP(G_50_‐S_50_)_20_‐DM1, due to the large number of S–S bonds in its structure, was designed to release DM1 in a controlled manner upon exposure to a reducing agent. Accordingly, the release of DM1 from hP(G_50_‐S_50_)_20_‐DM1 in PBS (pH = 7.4) and presence of glutathione (GSH) (10 mм) was investigated. The negative control experiment without GSH resulted in negligible (<5%) release of DM1 after 24 h, while the cumulative release indicated >96% release in the presence of GSH (Figure 6D). This excellent responsivity to GSH and specific release of drug was attributed to the high number of polysulfide segments in the structure of hP(G_50_‐S_50_)_20_‐DM1. After exploring the release profile of hP(G_50_‐S_50_)_20_‐DM1, the in vitro cytotoxicity of this system was investigated. hP(G_50_‐S_50_)_20_‐DM1 and free DM1 were incubated with MCF7 breast cancer cells, and the cell viability was evaluated after 24 h to assess their IC_50_ values. hP(G_50_‐S_50_)_20_‐DM1 exhibited an IC_50_ of 1.82 ng/mL, which is nearly 22‐fold lower than free DM1 with an IC_50_ of 39.2 ng/mL (Figure 6E). Importantly, hP(G_50_‐S_50_)_20_ did not show any significant toxicity alone towards MCF7 cells with concentrations ranging from 0.1 µg/mL to 0.1 mg/mL (Figure S4).

To determine the intracellular fate of the drug delivery system, the cellular uptake of hP(G_50_‐S_50_)_20_ labeled with Cy5 was studied using confocal laser scanning microscopy (CLSM). After 2 h of incubation with MCF7 cells, a clear fluorescence signal confirming the fast uptake of hP(G_50_‐S_50_)_20_ was observed (Figure 6F). This efficient copolymer uptake improves the delivery of conjugated DM1 to MCF7 cells.

## 4 Conclusion

We have developed a sustainable and scalable one‐pot method to synthesize hyperbranched polyglycerols with polysulfide linkages by copolymerizing glycidol and elemental sulfur, a major by‐product of the petroleum industry. The structural properties of the copolymers, including sulfur content and molecular weight, were tuned by varying the monomer feed ratio and initiator concentration. The polymerization mechanism involved nucleophilic attack by alkoxide and thiolate intermediates on glycidol and S_8_, followed by intramolecular rearrangement.

These sulfur‐containing copolymers showed high susceptibility to thiol–polysulfide exchange, enabling efficient conjugation and controlled release of the cytotoxic drug DM1. Importantly, DM1‐loaded copolymers exhibited significantly enhanced in vitro cytotoxicity, with IC_50_ values substantially lower than free DM1, demonstrating the therapeutic advantage of the delivery platform. Additionally, the polymers’ strong redox responsiveness positions them as promising candidates for targeted, stimulus‐responsive drug delivery.

## Author Contributions

**Paraskevi S. Stergiou**: synthesis, formal analysis, writing – original draft preparation; visualization. **Mariam Cherri**: experiment design. **Philip Nickl**: X‐ray spectroscopy measurements and data analysis. **Elisa Quaas**: cell viability and cellular uptake experiments. **Katharina Achazi**: design of cellular uptake and cell viability studies; confocal laser microscopy study, image analysis and interpretation. **Mathias Dimde**: cryogenic transmission electron microscopy images. **Rainer Haag, Mohsen Adeli**: conceptualization, supervision, resources, funding acquisition.

## Conflicts of Interest

The authors declare no conflict of interest.

## Supporting information




**Supporting File**: smll74499‐sup‐0001‐SuppMat.docx.

## Data Availability

The data that support the findings of this study are available from the corresponding author upon reasonable request.
