# Supplementary material for: Poly(Glycerol‐Sulfur) as a Functional Sustainable Nanomaterial: Synthesized by Anionic Ring Opening Polymerization of Elemental Sulfur
Source: Small. 2026 Jul 11;22(41):e74499. doi: 10.1002/smll.74499 (PMC13392745; doi:10.1002/smll.74499)
Supplement: Supplementary file 1 — Supporting File: smll74499‐sup‐0001‐SuppMat.docx. [file SMLL-22-e74499-s001.docx]

Supporting Information to:

**Poly(glycerol-sulfur) as a Functional Sustainable Nanomaterial: Synthesized by Anionic Ring Opening Polymerization of Elemental Sulfur**

Paraskevi S. Stergiou^1^, Mariam Cherri^1^, Philip Nickl^1^, Elisa Quaas^1^, Katharina Achazi^1^, Mathias Dimde^2^, Mohsen Adeli*^1,3^, Rainer Haag*^1^

^1^Institute of Chemistry and Biochemistry, Freie Universität Berlin, Takustr. 3, 14195 Berlin, Germany

^2^Institute of Chemistry and Biochemistry, Research Center of Electron Microscopy, Freie Universität Berlin, Berlin 14195, Germany

^3^Department of Chemistry, Lorestan University, Khorramabad, Iran

*Corresponding authors: m.aadeli@fu-berlin.de (M.A.); haag@chemie.fu-berlin.de (R.H.)

**Contents**

[Methods 3](#_Toc218785999)

[*Nuclear Magnetic Resonance (NMR)* 3](#_Toc218786000)

[*Elemental Analysis* 3](#_Toc218786001)

[*Raman Spectroscopy* 3](#_Toc218786002)

[*Thermogravimetric Analysis (TGA)* 3](#_Toc218786003)

[*X-Ray Photoelectron Spectroscopy* 4](#_Toc218786004)

[*Dynamic Light Scattering (DLS)* 4](#_Toc218786005)

[*High-Performance Liquid Chromatography (HPLC)* 4](#_Toc218786006)

[*Cell Viability Assay* 5](#_Toc218786007)

[*Cryogenic Transmission Electron Microscopy (cryo-TEM)* 6](#_Toc218786008)

[*Confocal Laser Scanning Microscopy (CLSM)* 6](#_Toc218786009)

[Synthesis 7](#_Toc218786010)

[***Synthesis of hP(Gx-Sy)z*** 7](#_Toc218786011)

[***Control reaction 1: Synthesis of hP(S_15_-G_85_)_5_*** 8](#_Toc218786012)

[***Control reaction 2: Synthesis of Polysulfur*** 8](#_Toc218786013)

[***Scale up synthesis: hP(G_85_-S_15_)_40_*** 9](#_Toc218786014)

[***Kinetic Study*** 9](#_Toc218786015)

[***Conjugation of DM1*** 10](#_Toc218786016)

[***Synthesis of Cy5-Maleimide*** 10](#_Toc218786017)

[***Conjugation of dye*** 11](#_Toc218786018)

[*XP survey spectra* 12](#_Toc218786019)

[*Calculation of the molecular weight of copolymers using ^1^H NMR spectra* 13](#_Toc218786020)

[*^13^C NMR spectra of the copolymers and sample calculation of the DB* 15](#_Toc218786021)

[*Raman Spectra of hP(G_85_-S_15_)_5_ in the region of 900-1500cm^-1^* 17](#_Toc218786022)

[*Determination of DM1 Encapsulation Efficiency and Release Profile of hP(G_50_-S_50_)_20_-DM1* 17](#_Toc218786023)

[Release Study 18](#_Toc218786024)

[*Cell Viability Assay of blank hP(G_50_-S_50_)_20_* 19](#_Toc218786025)

**Figures**

[**Figure S1.** A) Cy5-Maleimide synthetic pathway from Cy5. B) ^1^H NMR, and C) ^13^C NMR of Cy5-maleimide. 11](#_Toc199100268)

[**Figure S2.** XPS survey spectra of A) hP(G_85_-S_15_)_5_, B) hP(S_n_-G)_solid_, and C) polysulfur. 12](#_Toc199100269)

[**Figure S3.** ^1^H NMR spectra of A) hP(G_95_-S_5_)_5_, B) hP(G_50_-S_50_)_5_,C) hP(G_50_-S_50_)_10_, D) hP(G_50_-S_50_)_20_, and E) hP(G_85_-S_15_)_40_ (scale up reaction). 14](#_Toc199100270)

[**Figure S4.** Inverse-gated ^13^C-NMR of A) hP(G_95_-S_5_)_5_, B) hP(G_50_-S_50_)_5_, C) hP(G_50_-S_50_)_10_, D) hP(G_50_-S_50_)_20_, and E) hP(G_85_-S_15_)_40_ (scale up reaction). 15](#_Toc199100271)

[**Figure S5.** Raman spectra of hP(G_85_-S_15_)_5_, hPG, and S_8_, showing signals of oxidized sulfur species that indicate intramolecular rearrangement of C-S-O to more stable C-S=O bonds. 17](#_Toc199100272)

[**Figure S*6*.** Calibration curve of DM1 in ACN based on HPLC measurements. 18](#_Toc199100273)

[**Figure S7.** Cell viability assay of blank hP(G_50_-S_50_)_20_ showing no significant cell toxicity up to a concentration of 0.1 mg/mL. 19](#_Toc199100274)

**Tables**
[**Table S1.** Reaction conditions for polymer synthesis with different monomer ratios and molecular weights. 8](#_Toc216184642)

[**Table S2.** XPS-derived elemental composition of hP(G_85_-S_15_)_5_, hP(S_n_-G)_solid_, and polysulfur. 12](#_Toc216184643)

[**Table S3.** XPS peak-fit parameters for the C 1s and S 2p regions, including absolute binding energies, L-G mixing values, FWHM, asymmetry parameters, and absolute and relative areas of the samples hP(G_85_-S_15_)_5_, hP(S_n_-G)_solid_, and polysulfur. 12](#_Toc216184644)

[**Table S4.** Degree of branching and structural units’ abundance of hP(G_x_-S_y_)_z_. 16](#_Toc216184645)

# **Methods**

## *Nuclear Magnetic Resonance (NMR)*

NMR spectra were recorded on a Joel ECX 600 or on a Joel Eclipse 700 MHz spectrometer. Proton and carbon chemical shifts were given in ppm and referenced to the indicated solvents. Deuterated water (D_2_O) was the solvent of choice for synthesized polymer characterization, unless otherwise stated.

## *Elemental Analysis*

Elemental analysis was performed by a Vario EL CHNS element analyzer using Elementar Analysensysteme GmbH (Langenselbold, Germany).

## *Raman Spectroscopy*

The samples for characterization with Raman Spectroscopy were prepared via spin coating in a WS-650Mz-23NPPB model Spin Coater from Laurell Technologies, equipped with a 0523-101Q-G588DX model Rotary Vane Septic Air Pump from Gast. Each sample was coated on a ZYA quality Highly Oriented Pyrolytic Graphite (HOPG). For that, 20 μL of sample ( C = 100-200 mg/mL in MeOH) were placed on the HOPG layer with an Eppendorf pipette and the while was spin coated for 1 min, at 1500 rpm.

Raman spectra were recorded with an XploRA (Horiba) Raman spectrometer. All measurements were performed at 785 nm laser excitation with 25 μW laser power, an integration time of 1 s, an accumulation of 500 and at a 600 grating. A 100x objective (NA = 0.9) focused the laser beam on the sample. The Raman scattered light was detected in backscattering configuration by a Jobin-Yvon T64000 spectrometer in single mode (direct path) configuration. To detect the Raman scattered light, the spectrometer is equipped with an Andor iDus CCD camera. The intensity (integrated area under the peak) was plotted as a function of the excitation wavelength. To account for wavelength dependent changes in the sensitivity of the Raman setup, Raman intensity was calibrated on a silicon (Si/SiO_2_) wafer. All Raman measurements were performed under ambient conditions.

## *Thermogravimetric Analysis (TGA)*

TGA experiments were performed on the LINSEIS STA PTI600 (TG – DTA/DSC) machine in air atmosphere. The heating rate was set to 10 ^o^C / min and the temperature varied from 25 to 800 ^o^C. Calibration curves were measured for each sample. Measurements were performed in Al_2_O_3_ crucibles. The sample masses varied from 7 to 15 mg.

## *X-Ray Photoelectron Spectroscopy*

X-ray photoelectron spectroscopy experiments were performed with an EnviroESCA spectrometer (SPECS Surface Nano Analysis GmbH, Berlin, Germany), equipped with a monochromatic Al Kα X-ray source (Excitation Energy = 1486,71 electron volt (eV)) and a PHOIBOS 150 electron energy analyzer. The spectra were measured in normal emission, and a source-to-sample angle of 55° was used. All spectra were acquired in fixed analyzer transmission (FAT) mode. The binding energy scale of the instrument was calibrated, following a technical procedure provided by SPECS Surface Nano Analysis GmbH (calibration was performed according to ISO 15472). For quantification, the survey spectra were acquired with a pass energy of 80 eV, and the spectra were quantified utilizing the empirical sensitivity factors that were provided by SPECS Surface Nano Analysis GmbH (the sensitivity factors were corrected with the transmission function of the spectrometer). The highly resolved XP spectra were acquired with a pass energy of 50 eV, and the respective data were fitted using UNIFIT 2020 data processing software. For fitting, a Shirley background, and a Gaussian/Lorentzian sum function [peak shape model GL (30)] were used. If not denoted otherwise, the L-G mixing component was set to 0.30 for all carbon peaks and 0.40 for all heteroatom peaks. All binding energies were calibrated to the signal observed for the aliphatic C–C bond component (E_bind_ = 285 eV) if not denoted otherwise.

## *Dynamic Light Scattering (DLS)*

DLS measurements were performed in PBS and DMF with a Zetasizer Ultra by Malvern Instruments. Particle size was measured in UV-transparent disposable cuvettes filled with 80 µL solution. The samples were prepared at a concentration of 1 mg/mL. The samples were equilibrated for 1 min at 25°C; subsequently, the measurement was performed with 15 scans per sample for 3 independent measurements.

## *High-Performance Liquid Chromatography (HPLC)*

Mertansine was quantified by HPLC (Nexera series System Shimadzu, consisting of Pump LC-40D XR, Degasser DGU 403, Injector LH-40, Column Oven CTO-40S, Detector SPD M40), utilizing a Gemini 5μm C18 100A column (Phenomenex) maintained at 25°C. The solvent system used consisted of water (A) and acetonitrile (B), starting with 45% B for 5 minutes, going up to 90% B in 28 minutes, going down to 45% B in 2 minutes and holding 45% until 46 minutes total run time. Flow was maintained at 1 mL/min. Peaks were integrated at 232nm.

## *Cell Viability Assay*

Cell viability was determined using a CCK-8 Kit (Hycultec; Art. HY-K0301) according to the manufactor´s instructions. MCF7 (DSMZ no.: ACC 115) were cultured in Dulbecco’s Modified Eagle Medium (DMEM) supplemented with 10% (v/v) FBS, 100 U/mL penicillin and 100 μg/mL streptomycin. Cells were passaged every 3 to 4 days when reaching 70% to 90% confluency.

For the cell viability assay, the MCF7 cells were seeded in a 96-well plate at a density of 5 x 104 cells/mL in 90 µL DMEM Medium per well over night at 37°C and 5% CO_2_. 10 µL of sample (dissolved in deionized water) were added in serial dilutions including controls and incubated for another 24 h at 37°C and 5% CO_2_.

As controls non-treated cells and cells treated with 1% SDS were used. As solvent control, cells treated with 10% deionized water was used.

For background subtraction, wells containing no cells but only samples were used.

After 24h incubation the CCK8 solution was added (10 µL/well) and absorbance (450 nm / 650nm) was measured after approximately 3h incubation of the dye using a Tecan plate reader (SPARK, Tecan Group Ltd.). Measurements were performed in triplicates and repeated three times. The cell viability was calculated by setting the non-treated control to 100% and the non-cell control to 0% after subtracting the background signal using Excel software.

Cell viability (%) was plotted as a function of the drug concentration using OriginPro (OriginLab, Northampton, MA, USA). A new worksheet column was created containing the log₁₀-transformed concentrations, which was used as the X-axis for fitting. Dose–response curves were fitted using the “Dose–Response (log[inhibitor] vs. response - Variable Slope)” function in Origin’s Nonlinear Curve Fit module, following a four-parameter logistic (4PL) model. The IC₅₀ values, corresponding to the drug concentration that reduces cell viability by 50%, were calculated from the fitted logX₀ parameters. For the two conditions tested, IC₅₀ values were 1.82 nM and 39.2 nM, respectively. Each concentration point was measured in triplicate, and IC₅₀ values are reported as mean ± standard deviation from at least three independent experiments.

## *Cryogenic Transmission Electron Microscopy (cryo-TEM)*

Perforated carbon film-covered microscopical 200 mesh grids (R1/4 batch of Quantifoil, MicroTools GmbH, Jena, Germany) were cleaned with chloroform and hydrophilized by 60 s glow discharging at 10 mA in a Safematic CCU-010 device (safematic GmbH, Zizers, Switzerland). Subsequently, 4 μL aliquots of the sample solution were applied to the grids. The samples were vitrified by automatic blotting and plunge freezing with a FEI Vitrobot Mark IV (Thermo Fischer Scientific Inc., Waltham, Massachusetts, USA) using liquid ethane as cryogen.

The vitrified specimens were transgerred to the autoloader of a FEI TALOS ARCTICA electron microscope (Thermo Fisher Scientific Inc., Waltham, Massachusetts, USA). This microscope is equipped with a high-brightness field-emission gun (XFEG) operated at an acceleration voltage of 200 kV. Micrographs were acquired on a FEI Falcon 3 direct electron detector (Thermo Fisher Scientific Inc., Waltham, Massachusetts, USA) using a 100 μm objective aperture.

## *Confocal Laser Scanning Microscopy (CLSM)*

Cellular uptake of polymer labeled with Cy5 in MCF7 cancer cells (DSMZ no.: ACC 115) was monitored by confocal laser scanning microscopy (CLSM). The cells were cultured as described above. For cLSM, 270 µL of cells in DMEM were seeded in each well of an 8-well ibidi μ-slides (50.000 cells mL^-1^). After 1 day, 30 µL of polymer were added at a final test concentration of 1 mg mL^-1^. The cells were incubated for 24 hours. 1 hour before the imaging, cell nuclei were stained with 1 μg mL^-1^ Hoechst 33342 (Life Technologies GmbH, Darmstadt, Germany). Then confocal images were taken by using an inverted confocal laser scanning microscope Leica DMI6000CSB SP8 (Leica, Wetzlar, Germany) with a 63x/1.4 HC PL APO CS2 oil immersion objective and the LAS X software.

Brightness and Contrast have been adjusted using Fiji using a macro. In short, for the brightfield and nuclei Hoechst 33442 channel, the auto adjustment was used; for the Cy5 channel, the brightness and contrast were adjusted to a minimum of 13 and a maximum 66.

**Materials**

Glycidol 96.0% pure, Sulfur and 1,1,1-Tris(hydroxymethyl)propane dist. > 98.0% pure from Sigma-Aldrich, Potassium Hydroxide from Fischer Scientific, and Methanol, 99.8%, extra dry over Molecular Sieve from Acros Organic were used for the polymer synthesis. Glycidol was distilled prior the employment. DM1 from MedChem Express was used for the drug conjugation.

# **Synthesis**

***Synthesis of hP(Gx-Sy)z.*** The synthesis of hyperbranched polyglycerol with oligosulfur segments in its scaffold was carried out in a 100 mL 3-neck Schlenck flask, equipped with a magnetic stirring bar in the case of 5 kDa MW polymers, or a mechanical stirrer in the case of 10 kDa or higher MW polymers. The reaction was carried out at specific temperature, under argon and at a stirring speed of 100 rpm. Trimethylolpropane (*Table 5*) was melted under inert atmosphere at 65 ^o^C and then was dried under vacuum for 30 min. The temperature was then set at 55 ^o^C and a mixture of dry MeOH (0.25 mL) and KOH (40% of TMP) was added to the Schlenck flask under argon and was let to deprotonate TMP for 1h. The excess MeOH was subsequently removed under vacuum for 1h. Glycidol (5.5g, 5 mL, 1eq) was added dropwise via a syringe pump (2.5 mL/h) under argon, and the temperature was set at 100 ^o^C. After 1h of glycidol addition, elemental sulfur (*Table 5*) was added into the reaction mixture and the temperature was set at 120 ^o^C. After ca. 20 min, elemental sulfur melted completely, and the reaction was let to stir at 120^o^C overnight. The reaction was quenched with MeOH and was let to cool down. The reaction mixture was filtrated through a filter paper to remove unreacted elemental sulfur, and the liquid product was then concentrated under reduced pressure and purified through precipitation in cold acetone. Finally, the precipitate was dissolved in distilled water and transferred in a preweighted vial, which was lyophilized to afford the product.

Although the formula of elemental sulfur was described as S_8_, its quantities used in the reactions are always given in atomic equivalents (32 g/atom) in the interest of clarity and to avoid confusion.

**Table S1.** Reaction conditions for polymer synthesis with different monomer ratios and molecular weights.

| **Experiment** | **Gly (mL/eq)** | **S_8_ (eq)** | **TMP (eq)** | **α (%)** |
| --- | --- | --- | --- | --- |
| **hP(G_95_-S_5_)_5_** | 5/1 | 1:24 | 1:62 | 79 |
| **hP(G_85_-S_15_)_5_** | 5/1 | 1:6 | 1:62 | 78 |
| **hP(G_50_-S_50_)_5_** | 5/1 | 1 | 1:47 | 30 |
| **hP(G_50_-S_50_)_10_** | 5/1 | 1 | 1:94 | 39 |
| **hP(G_50_-S_50_)_20_** | 5/1 | 1 | 1:189 | 25 |

***Control reaction 1: Synthesis of hP(S_15_-G_85_)_5_.*** In a 100 mL 3-neck Schlenck flask, equipped with a magnetic stirring bar, TMP (174.4 mg, 1.3 mmol, 1/47 eq) was melted under inert atmosphere at 65 ^o^C and then was dried under vacuum for 30 min. The temperature was then set at 55 ^o^C and a mixture of dry methanol (0.5 mL) and potassium hydroxide (29 mg, 0.52 mmol) was added to the Schlenck flask under argon and was let to deprotonate TMP for 1h. The excess methanol was subsequently removed under vacuum for 1h. The temperature was set to 120 ^o^C, and elemental sulfur (1.952 g, 0.061 mol, 1 eq) was added under argon and was let to melt. After 1h, glycidol (5 mL, 1 eq) was added dropwise via a syringe pump (2.5 mL/h) under argon and the reaction was let to stir at 120 ^o^C overnight. The next day, a brown solid product along with a brown liquid product were observed. The reaction was quenched with MeOH, and the solid product was filtered out with a filter paper and characterized by Elemental Analysis, Raman Spectroscopy and XPS. The liquid product was concentrated under reduced pressure and purified by precipitation in cold acetone. The final product was characterized by NMR, Elemental Analysis, Raman Spectroscopy and XPS.

***Control reaction 2: Synthesis of Polysulfur.*** In a 100 mL Schlenck flask, equipped with a magnetic stirring bar, TMP (107 mg, 0.8 mmol, 1/156 eq) was added and melted under argon at 65 ^o^C. Subsequently, TMP was dried under vacuum for 30 min. The temperature was then set at 55 ^o^C and a mixture of dry methanol (0.3 mL) and potassium hydroxide (18 mg, 0.32 mmol) was added to the Schlenck flask under argon and was let to deprotonate TMP for 1h. The excess MeOH was subsequently removed under vacuum for 1h. The temperature was then set to 120 ^o^C, and elemental sulfur (4 g, 0.125 mol, 1 eq) was added under argon. The reaction was let to stir at 120 ^o^C overnight. The next day, a yellow solid product was formed, which was unable to be purified since it was insoluble in everything. The product was characterized by Raman Spectroscopy, X-Ray Photoelectron Spectroscopy (XPS).

***Scale up synthesis: hP(G_85_-S_15_)_40_*.** The scale up synthesis was carried out in a 1 L cylindrical round bottom reaction vessel equipped with an external mechanical stirrer. TMP (164 mg, 1.2 mmol, 1/498) was melted at 60 ^o^C for 45 min and the excess water was then removed under high vacuum for 1h. The temperature was set to 50 ^o^C and a mixture of potassium hydroxide (27 mg, 0.48 mmol) in dry methanol (0.5 mL) was added under vacuum and was let to deprotonate TMP for 1h. The methanol formed was removed under high vacuum for 1h. The temperature was then set to 70 ^o^C and glycidol (50 mL, 1eq) was added via a syringe pump (rate: 10 mL/h). When 25 mL of glycidol had been added, elemental sulfur (3.91 g, 1/6 eq) was also added, and the temperature was set at 120 ^o^C. The rest of glycidol was poured in the reaction mixture, and the whole was let to stir at 120 ^o^C, 100 rpm, overnight. The reaction was quenched with methanol. For the purification, the reaction mixture was first filtered through filter paper and precipitated in cold acetone. The supernatant was discarded, and the precipitate was redissolved in water and lyophilized.

***Kinetic Study.*** The kinetic study was carried out following the usual synthesis procedure of hP(G_85_-S_15_)_5_, targeting a 6:1 (Gly:S_8_) monomer ratio and a 5 kDa molecular weight. Trimethylolpropane (131 mg, 0.98 mmol, 1/62 eq) was melted under inert atmosphere at 65 ^o^C and then was dried under vacuum for 30 min. The temperature was then set at 55 ^o^C and a mixture of dry methanol (0.4 mL) and potassium hydroxide (22 mg) was added to the Schlenck flask under argon and was let to deprotonate TMP for 1h. The excess methanol was subsequently removed under vacuum for 1h. Glycidol (5 mL, 0.074 mol, 1 eq) was added dropwise via a syringe pump (2.5 mL/h) under argon, and the temperature was set at 100 ^o^C. Aliquots were taken after 30 min and 1h, which was right before the addition of S_8_. After that, S_8_ ( 385 mg, 0.012 mol, 1/6 eq) was added and glycidol kept being poured into the reaction mixture. More aliquots were collected 30 min, 1 h, 2 h, 3 h, 4 h and 24 h after elemental sulfur’s addition. Each aliquot was collected with the help of an Eppendorf tip, was placed in a preweighted vial, and was immersed in an ice bath to quench the reaction. Once the reaction was completed, it was quenched with methanol and followed the work up us described in the polymer synthesis above. All aliquots were characterized by *^1^*H NMR, in a mixture of deuterated chloroform : methanol (4:1).

***Conjugation of DM1*.** Drug conjugation was achieved via nanoprecipitation. A 1.84 mL solution of premixed hP(G_50_-S_50_)_20_ (1.8 mL, 20 mg/mL) and DM1 (0.4 mL, 10 mg/mL) in dimethylformamide (DMF) was added dropwise into 15.8 mL of distilled water under intense stirring at 1200 rpm and then was stirred at 200 rpm for 24 hours. The solution was dialyzed (MWCO = 3.5 kDa) against distilled water for 48 hours and lyophilized. The size and polydispersity of hP(G_50_-S_50_)_20_-DM1 were determined by dynamic light scattering (DLS) (Figure 6C).

***Synthesis of Cy5-Maleimide.*** Cy5-Acid (24.0 mg, 42.6 µmol, 1.0 eq.) was dissolved in DMF (500 µL) at ambient temperature. HATU (21.1 mg, 55.4 µmol, 1.3 eq.) and Et 3 N (34.5 mg, 47.3 µL, 314 µmol, 8.0 eq.) were added subsequently and the reaction mixture was stirred for 5 min. Then, amine (12.1 mg, 68.2 µmol, 1.6 eq.) was added and the reaction was stirred overnight. After precipitation and decantation from Et_2_O (45 mL), the crude product was purified by automated flash chromatography (dry-loaded on Isolute®, SiO_2_, DCM/MeOH 0% to 20%) and subsequent preparative HPLC (preparative column: Phenomenex Gemini-NX-C18, 5 µm, 250 x 30 mm, Solvent A: H_2_O/MeCN 95:5, solvent B: H_2_O/MeCN 5:95 – 10 min 100:0 isocratic, 11-30 min 70:30 isocratic).

Analytical HPLC determined the purity of collected fractions. Cy5-Maleimide (9.70 mg, 14.5 µmol, 34%) was obtained as a blue solid.

^1^H NMR (CD_3_OD, 600 MHz): δ = 8.35 – 8.25 (m, 1H), 8.23 (t, J = 13.1, 1H, major), 8.23 (t, J = 13.0 Hz, 1H, minor), 7.87 – 7.84 (m, 2H), 7.53 (dd, J = 7.5, 1.2 Hz, 1H), 7.44 (td, J = 7.7, 1.2 Hz, 1H), 7.39 – 7.34 (m, 1H), 7.32 (td, J = 7.4, 0.9 Hz, 1H), 7.30 – 7.25 (m, 1H), 6.80 (s, 2H, major), 6.66 (t, J = 12.4 Hz, 1H, minor), 6.65 (t, J = 12.4 Hz, 1H, major), 6.46 – 6.35 (m, 2H, major), 6.28 – 6.22 (m, 1H), 6.21 – 6.12 (m, 1H, minor), 5.98 – 5.81 (m, 1H, minor), 4.59 (s, br, 1H), 4.33 – 4.24 (m, 2H, minor), 4.07 (t, J = 7.5 Hz, 2H), 3.75 – 3.72 (m, 2H, minor), 3.69 (s, 3H, minor), 3.68 (s, 3H, major), 3.66 – 3.62 (m, 4H), 3.61 – 3.57 (m, 2H, major), 3.36 – 3.33 (m, 2H, major), 2.21 (t, J = 7.3 Hz, 2H, minor), 2.13 (t, J = 7.3 Hz, 2H, major), 1.86 – 1.76 (m, 2H), 1.75 – 1.72 (m, 12H), 1.72 – 1.60 (m, 2H), 1.48 – 1.39 (m, 2H) ppm.


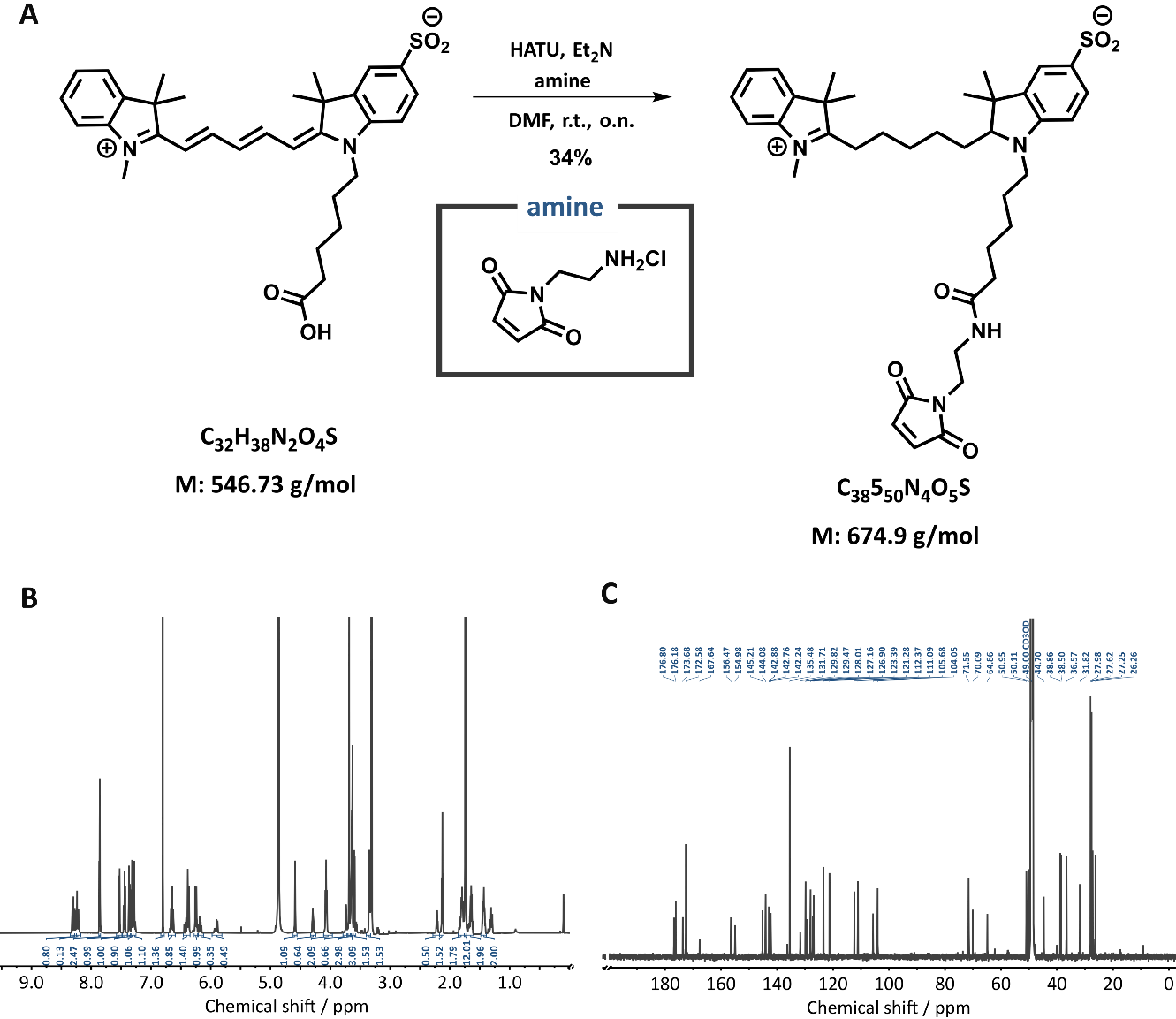


**Figure S1.** A) Cy5-Maleimide synthetic pathway from Cy5. B) ^1^H NMR, and C) ^13^C NMR of Cy5-maleimide.

***Conjugation of dye.*** hP(G_50_-S_50_)_20_ (1.8 mL, 20 mg/mL) and Cy5-maleimide (0.2 mL, 10 mg/mL) both dissolved in DI water were stirred at 150 rpm overnight. The next day, the product was purified through a preswollen LH-25 Sephadex column and the product was lyophilized.

# ***XP survey spectra***


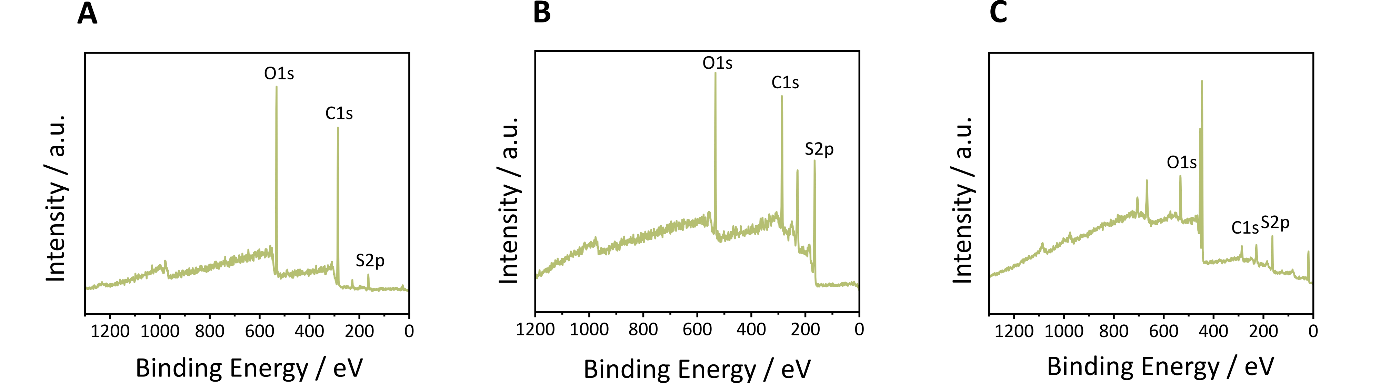

**Figure S2.** XP survey spectra of A) hP(G_85_-S_15_)_5_, B) hP(S_n_-G)_solid_, and C) polysulfur.

**Table S2.** XPS-derived elemental composition of hP(G_85_-S_15_)_5_, hP(S_n_-G)_solid_, and polysulfur.

| Sample name | C (%) | O (%) | S (%) |
| --- | --- | --- | --- |
| hP(G_85_-S_15_)_5_ | 64.98 ± 0.61 | 31.43 ± 0.58 | 3.59 ± 0.27 |
| hP(S_n_-G)_solid_ | 49.65 ± 0.97 | 19.62 ± 0.65 | 30.72 ± 0.75 |
| PS | 27.32 ± 3.63 | 9.30 ± 1.60 | 63.37 ± 3.43 |

**Table S3.** XPS peak-fit parameters for the C 1s and S 2p regions, including absolute binding energies, L-G mixing values, FWHM, asymmetry parameters, and absolute and relative areas of the samples hP(G_85_-S_15_)_5_, hP(S_n_-G)_solid_, and polysulfur.

| Sample name | Peak | Binding Energy (eV) | Peak Assignment | L-G mixing | FWHM (eV) | Asymmetry | Abs. Area | Rel. Area (%) |
| --- | --- | --- | --- | --- | --- | --- | --- | --- |
| hP(G_85_-S_15_)_5_ | C1s | 285.03 | C-C, C-S | 0.3 | 1.25 | 0 | 899.58 | 0.18 |
|  |  | 286.44 | C-O | 0.3 | 1.25 | 0 | 4144 | 0.82 |
|  | S2p_3/2_ | 163.73 | C-S, S-S | 0.4 | 1.29 | 0 | 287.92 | 0.66 |
| hP(S_n_-G)_solid_ | C1s | 284.97 | C-C, C-S | 0.3 | 1.3 | 0 | 742.4 | 0.11 |
|  |  | 285.85 | C-O | 0.3 | 1.3 | 0 | 2203 | 0.33 |
|  |  | 286.96 | C-S=O-R | 0.3 | 1.3 | 0 | 3159 | 0.47 |
|  |  | 288.15 | C-S(=O)_2_-R | 0.3 | 1.3 | 0 | 601.8 | 0.09 |
|  | S2p_3/2_ | 167.65 | C-S, S-S | 0.4 | 1.3 | 0 | 582.8 | 0.082 |
|  |  | 165.47 | C-S=O-R | 0.4 | 1.3 | 0 | 2433 | 0.34 |
|  |  | 164.45 | C-S(=O)_2_-R | 0.4 | 1.3 | 0 | 1741 | 0.24 |
| PS | C1s | 285.00 | C-C, C-S | 0.3 | 1.4 | 0 | 85.86 | 0.20 |
|  |  | 286.18 | C-O | 0.3 | 1.4 | 0 | 280.23 | 0.66 |
|  |  | 287.64 | C-S=O-R | 0.3 | 1.4 | 0 | 57.42 | 0.14 |
|  | S2p_3/2_ | 167.33 | C-S, S-S | 0.4 | 1.5 | 0 | 98.41 | 0.03 |
|  |  | 165.87 | C-S=O-R | 0.4 | 1.5 | 0 | 1388 | 0.49 |
|  |  | 163.77 | C-S(=O)_2_-R | 0.4 | 1.5 | 0 | 404.15 | 0.14 |

# ***Calculation of the molecular weight of copolymers using ^1^H NMR spectra***

The molecular weight of the copolymers was calculated by first integrating the peak at 0.9 ppm to the 3 methyl protons of TMP, followed by the subsequent integration of the peaks between 3.4 - 4.2 ppm which correspond to the 5 protons of polyglycerol. By dividing the relative integral of these signals by the 5 protons, we calculated the number of the repeating units. Multiplying the repeating units by the molecular weight of each repeating unit reveals the molecular weight of the copolymer (Equation S1).

$Molecular Weight \left( \frac{g}{mol} \right)=\frac{\int\left( 3.4-4.2ppm \right)}{5 protons}x(74$) (1)

*Sample calculation for hP(G_50_-S_50_)_20_ based on Equation S2-7:*

$$Molecular Weight \left( \frac{g}{mol} \right)=\frac{1530.01}{5}x74=22644.15\frac{g}{mol}=22.6 kDa$$

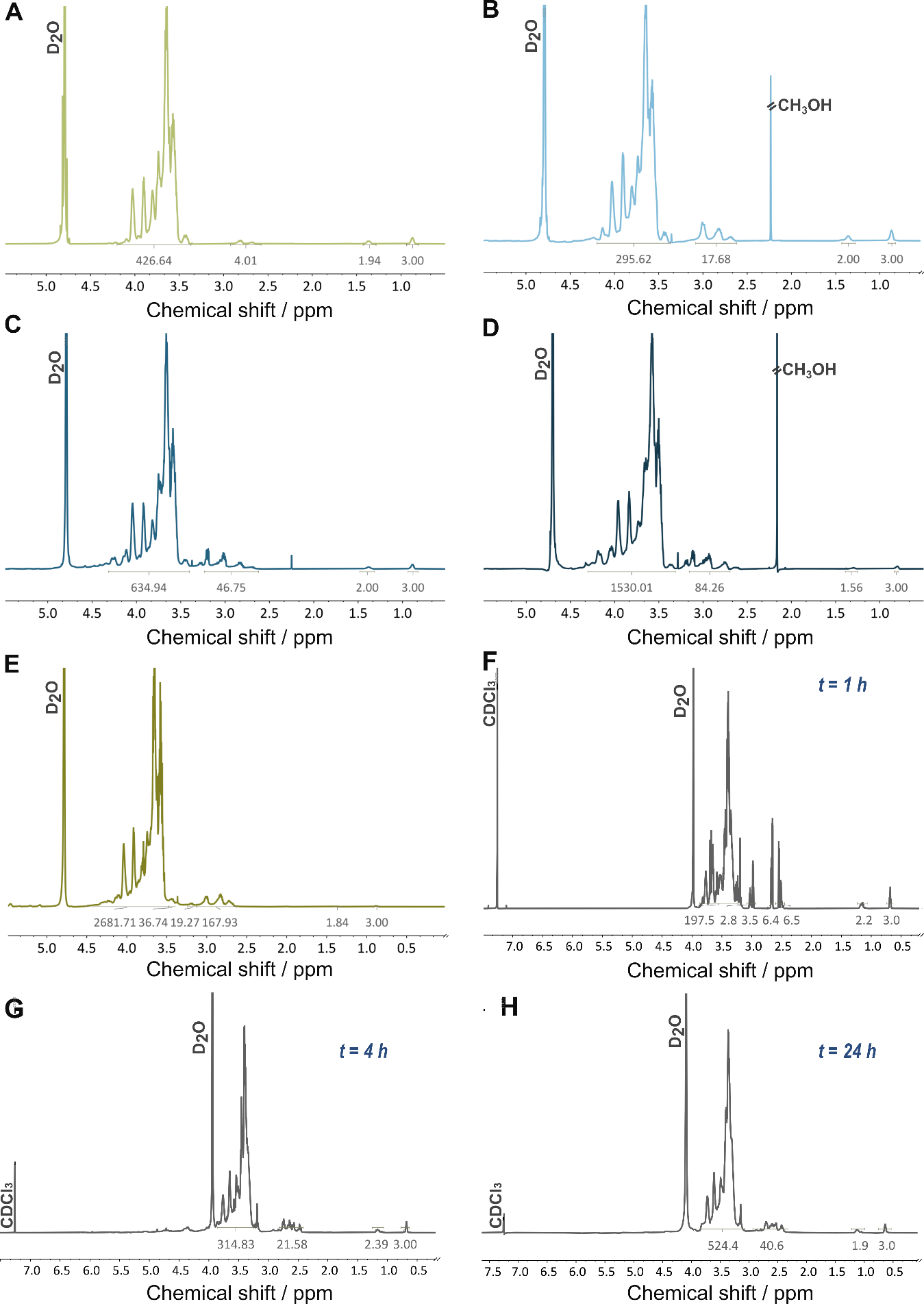

**Figure S3.** ^1^H NMR spectra of A) hP(G_95_-S_5_)_5_, B) hP(G_50_-S_50_)_5_,C) hP(G_50_-S_50_)_10_, D) hP(G_50_-S_50_)_20_, E) hP(G_85_-S_15_)_40_ (scale up reaction) all recorded in D_2_O. (F–H) Time-resolved ^1^H NMR spectra obtained from aliquots during the kinetic study of hP(G_85_–S_15_)_5_: (F) t = 1 h (prior to S₈ addition), (G) t = 4 h, and (H) t = 24 h, all recorded in a mixture of CDCl_3_:D_2_O (4:1).

# ***
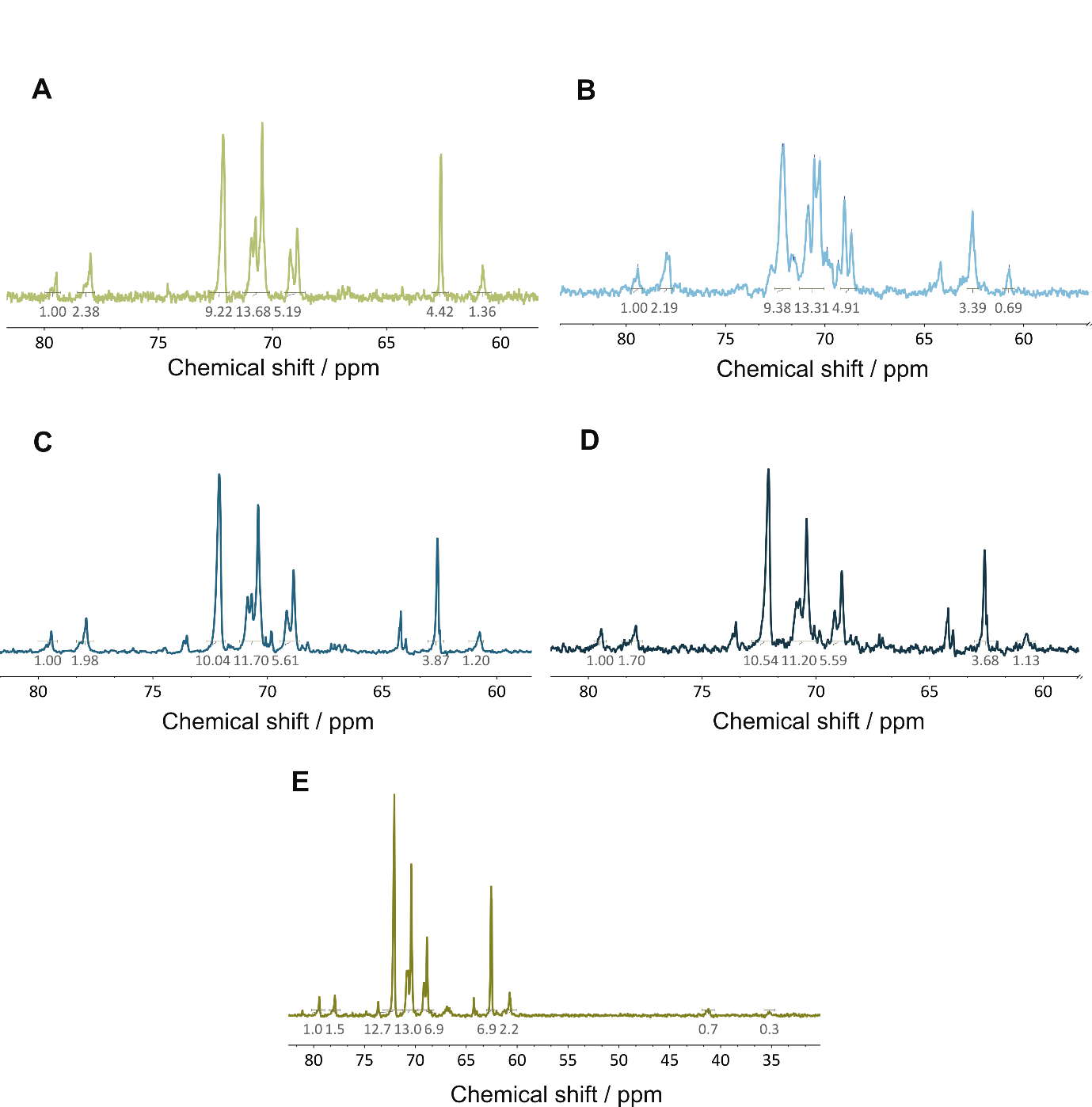
^13^C NMR spectra of the copolymers and sample calculation of the DB***

**Figure S4.** Inverse-gated ^13^C-NMR of A) hP(G_95_-S_5_)_5_, B) hP(G_50_-S_50_)_5_, C) hP(G_50_-S_50_)_10_, D) hP(G_50_-S_50_)_20_, and E) hP(G_85_-S_15_)_40_ (scale up reaction).

The structural units’ relative abundance and the *DB* were calculated using the method reported by Frey et al.^[1,2]^

The structural units’ relative abundance was calculated by dividing the relative integrals of the corresponding unit by the sum of the relative integral of all units (*Equation S2-7*).

Sum of all units’ integrals = L_1,3_$\int60-61$ + ½ L_1,4_ $\int72-73.5$ + D $\int77.5-79$ + T $\int62-63$ ( 2 )

$L1,3=\frac{L1,3\int60-61}{sum of all units^{'}integrals}$ ( 3 )

$L1,4=\frac{\frac{1}{2}L1,4\int72-73.5}{sum of all units^{'}integrals}$ ( 4 )

$D=\frac{D\int77.5-79}{sum of all units^{'}integrals}$ ( 5 )

$T=\frac{T\int62-63}{sum of all units^{'}integrals}$ ( 6 )

The degree of branching can be calculated from each fraction of the structural units respectively, as shown in *Equation 7*.

$DB =\frac{2D}{2D+L1,3+L1,4}$ ( 7 )

**Table S4.** Degree of branching and structural units’ abundance of hP(G_x_-S_y_)_z_.

|  |  | Relative Integral | | | | |  |
| --- | --- | --- | --- | --- | --- | --- | --- |
|  | **Shift (ppm)** | **hP(G_95_-S_5_)_5_** | **hP(G_85_-S_15_)_5_** | **hP(G_50_-S_50_)_5_** | **hP(G_50_-S_50_)_10_** | **hP(G_50_-S_50_)_20_** | **hP(G_85_-S_15_)_40_** |
| L_1,3_ | 79-80 | 1.00 | 1.00 | 1.00 | 1.00 | 1.00 | 1.00 |
| D | 77.5-79 | 2.30 | 2.15 | 2.19 | 2.02 | 1.70 | 1.45 |
| 2L_1,4_ | 72-73.5 | 9.57 | 7.25 | 9.38 | 10.11 | 10.54 | 12.71 |
| 2D, 2T | 70-71.5 | 13.84 | 11.46 | 13.31 | 11.79 | 11.20 | 13.02 |
| L_1,3_, L_1,4_ | 68.5-70 | 4.49 | 4.47 | 4.91 | 5.61 | 5.59 | 6.87 |
| T | 62-63 | 4.05 | 3.83 | 3.39 | 3.87 | 3.68 | 6.86 |
| L_1,3_ | 60-61 | 0.98 | 1.06 | 0.69 | 1.13 | 1.13 | 2.15 |
|  |  | **Relative Abundance (%)** | | | | |  |
| T units |  | 33 | 36 | 31 | 32 | 31 | 41 |
| D units |  | 19 | 20 | 20 | 17 | 14 | 9 |
| L_1,3_ units |  | 8 | 10 | 6 | 9 | 10 | 6 |
| L_1,4_ units |  | 39 | 34 | 43 | 42 | 45 | 38 |
| *DB* |  | 0.44 | 0.48 | 0.45 | 0.39 | 0.37 | 0.29 |

*Sample calculation for hP(G_50_-S_50_)_20_ based on Equation S2-7:*

Sum of all units’ integrals = *L*_1,3_$\int60-61$ + ½ *L*1,4 $\int72-73.5$ + D $\int77.5-79$ + T $\int62-63$

$$L1,3=\frac{1.13}{1.13+\frac{1}{2} 10.54+1.70+3.68}=0.1$$

$$L1,4=\frac{\frac{1}{2}10.54}{1.13+\frac{1}{2} 10.54+1.70+3.68}=0.45$$

$$D=\frac{1.70}{1.13+\frac{1}{2} 10.54+1.70+3.68}=0.14$$

$$T=\frac{3.68}{1.13+\frac{1}{2} 10.54+1.70+3.68}=0.31$$

$$DB=\frac{2x0.14}{2x0.14+0.1+0.45}=0.37$$

# ***Raman Spectra of hP(G_85_-S_15_)_5_ in the region of 900-1500cm^-1^***

**
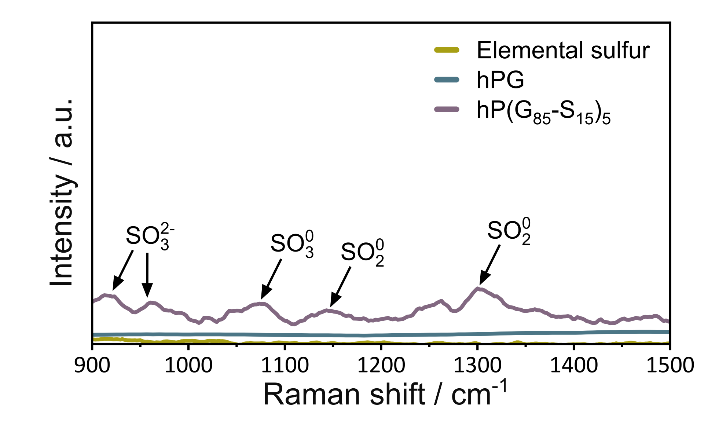
**


**Figure S5.** Raman spectra of hP(G_85_-S_15_)_5_, hPG, and S_8_, showing signals of oxidized sulfur species that indicate intramolecular rearrangement of C-S-O to more stable C-S=O bonds.

# ***Determination of DM1 Encapsulation Efficiency and Release Profile of hP(G_50_-S_50_)_20_-DM1***

For the determination of DM1 content, 0.1 mL of hP(G_50_-S_50_)_20_-DM1 (10 mg/mL) in distilled water was added in a solution of 100 mM DTT (2.9 mL) and was stirred for 96 hours to degrade completely. The sample was eventually lyophilized, redissolved in 100 μL of DMF, and subjected to high-performance liquid chromatography measurements for DM1 quantification, based on an HPLC-based calibration curve of DM1 in DMF (Figure S5). The loading capacity (LC) and loading efficiency (LE) of the drug were calculated according to the following formulas.

$$LC(wt\%)=\frac{weight of initial drug-weight of encapsulated drug}{total weight of polymer and drug obtained}x100$$

$$LE(\%)=\frac{weight of loaded drug}{weight of initial drug}x100$$

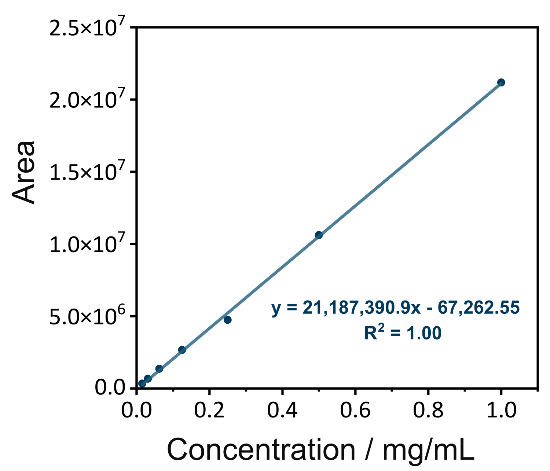


**Figure S*6*.** Calibration curve of DM1 in ACN based on HPLC measurements.

# **Release Study**

For the release study, 1 mg of hP(G_50_-S_50_)_20_ was dissolved in 0.8 mL of either 10 mM GSH in PB (pH = 7.4), or PB (pH = 7.4) as a control, and placed in 3500 Da midi pur-a-lyzers. The pur-a-lyzers were then placed in 50 mL falcon tubes which were filled with 20 mL of the respective medium and were immediately incubated at 37 ^o^C while shaking at 200 rpm. After t = 1, 2, 4, 6, 8, 12, and 24 h, 5 mL from each falcon tube was placed in a 10 mL vial and lyophilized, while 5 mL of fresh medium was replaced in the falcon tube, resulting always in a 20 mL volume. After the samples were lyophilized, they were redissolved in 200 μL MeCN/H_2_O (55:45) and submitted for HPLC measurements. Based on a standard calibration curve of DM1 (Figure S5), the amount of DM1 released after each time frame was identified and the cumulative release was calculated. The release study was conducted in triplicates.

# ***Cell Viability Assay of blank hP(G_50_-S_50_)_20_***

***
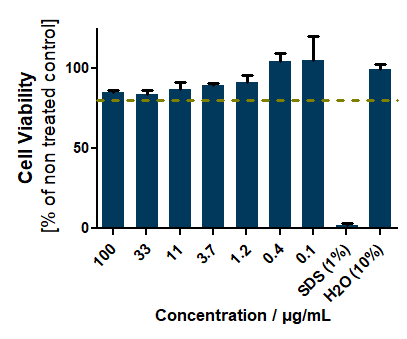
***

**Figure S7.** Cell viability assay of blank hP(G_50_-S_50_)_20_ showing no significant cell toxicity up to a concentration of 0.1 mg/mL.
